# A Chimera of Th1 Stimulatory Proteins of *Leishmania donovani* Offers Moderate Immunotherapeutic Efficacy with a Th1-Inclined Immune Response against Visceral Leishmaniasis

**DOI:** 10.1155/2021/8845826

**Published:** 2021-02-27

**Authors:** Sneha Ratnapriya, Keerti, Narendra Kumar Yadav, Anuradha Dube, Amogh Anant Sahasrabuddhe

**Affiliations:** ^1^Molecular and Structural Biology Division, CSIR-Central Drug Research Institute, Lucknow 226031, India; ^2^Molecular Parasitology and Immunology Division, CSIR-Central Drug Research Institute, Lucknow 226031, India

## Abstract

Immunotherapy, a treatment based on host immune system activation, has been shown to provide a substitute for marginally effective conventional chemotherapy in controlling visceral leishmaniasis (VL), the deadliest form of leishmaniasis. As the majority of endemic inhabitants exhibit either subclinical or asymptomatic infection which often develops into the active disease state, therapeutic intervention seems to be an important avenue for combating infections by stimulating the natural defense system of infected individuals. With this perspective, the present study focuses on two immunodominant *Leishmania* (*L.*) *donovani* antigens (triosephosphate isomerase and enolase) previously proved to be potent prophylactic VL vaccine candidates, for generating a recombinant chimeric antigen. This is based on the premise that in a heterogeneous population, a multivalent antigen vaccine would be required for an effective response against leishmaniasis (a complex parasitic disease). The resulting molecule rLdT-E chimeric protein was evaluated for its immunogenicity and immunotherapeutic efficacy. A Th1 stimulating adjuvant BCG was employed with the protein which showed a remarkable 70% inhibition of splenic parasitic multiplication positively correlated with boosted Th1 dominant immune response against lethal *L. donovani* challenge in hamsters as evidenced by high IFN-*γ* and TNF-*α* and low IL-10. In addition, immunological analysis of antibody subclass presented IgG2-based humoral response besides considerable delayed-type hypersensitivity and lymphocyte proliferative responses in rLdT-E/BCG-treated animals. Our observations indicate the potential of the chimera towards its candidature for an effective vaccine against *Leishmania donovani* infection.

## 1. Introduction

Visceral leishmaniasis (VL), a fatal protozoan disease, commonly affects poor communities of the world with prevalence in India, Brazil, Sudan, South Sudan, Ethiopia, Kenya, and Somalia [1]. Though there is a reduction in VL cases in recent years due to the available chemotherapeutics, management of asymptomatic/subclinical infection has become an increasingly major challenge for the disease control program [2]. Moreover, the expansion of HIV-VL coinfections as well as post-kala-azar dermal leishmaniasis (PKDL) cases has severely influenced the VL epidemiology [3, 4]. In these circumstances, wherein the immunity of personnel is suppressed, there is an obligatory need for the development and implementation of therapeutic intervention that could aid in restoring or promoting an effective immune response with resolution of infection and long-lasting protection, hence presenting a better alternative to chemotherapy. So far, there are not many studies on VL immunotherapy; however, cutaneous leishmaniasis (CL) has a long history with some promising results for first- and second-generation vaccines [5]. Immunotherapy using vaccine combined with potential immunomodulators can be an effective modality to treat VL, and there is not any possibility for resistance development with this intervention [6].

Despite enormous advancement being made in vaccine development against VL, there is no licensed vaccine for humans so far, and it is uncertain that a single recombinant protein could provide the required level of protection against the complex parasite in a genetically polymorphic host system, thus leading to the advent of the chimeric vaccine concept. Of note, till date, the most evolved prophylactic or therapeutic vaccine candidates intended to control human leishmaniasis rely upon recombinant multiantigen molecules [7–13]. These kinds of chimeric vaccines consisting of more than one distinct *Leishmania* antigen molecule could be more economical to produce and show good manufacturing practices and essentially highly immunogenicity due to the presence of multiple T-cell epitopes in a single vaccine. Nonetheless, the success of therapeutic vaccine candidates against CL and MCL in Phase II trials [14, 15] showed a ray of hope towards developing the immunotherapeutic vaccine against human VL too. It is apparent that these subunit vaccines with strong adjuvants could be satisfactorily immunogenic, and such vaccination approach would take us closer to the realization of a safe and cost-effective vaccine against *Leishmania* spp. as well.

Previously, several proteins from the soluble fraction of the promastigote stage were found to have Th1 stimulatory potential [16, 17] of which enolase and triosephosphate isomerase (TPI) were reported to confer considerable prophylactic efficacy and Th1 skewed immune response against *Leishmania* challenge [18, 19]. These two vital glycolytic enzymes have been considered potential vaccine targets in other diseases as well [20–22]. In the present investigation, we generated a single recombinant chimeric protein comprising the sequences of enolase and TPI genetically linked in tandem and characterized its possible immunotherapeutic effect on *L. donovani*-infected hamsters with the aim of contributing towards the design and development of an effective vaccine against visceral leishmaniasis.

## 2. Methodology

### 2.1. Animal and Parasite

Female Syrian golden hamsters (*Mesocricetus auratus*, 5–6 weeks old) were used as the experimental host, which was maintained in the laboratory animal facility of CSIR-CDRI. The care and use of hamsters were monitored by the institutional animal ethics committee (IAEC) which also approved animal experimentation protocols following the guidelines of the committee for control and supervision of experiments on animals (CPCSEA). *Leishmania* (*L.*) *donovani* strain (MHOM/IN/80/Dd8) was procured as promastigotes from American Type Culture Collection (ATCC, Manassas, VA, USA) and maintained *in vitro* following the protocol of Garg et al. [23]. Soluble *L. donovani* (SLD) was prepared from promastigotes, quantified by the Bradford assay, and aliquots were kept at -80°C until further use [24].

### 2.2. Production of the Immunogen: Recombinant Chimeric Protein

The chimeric gene was constructed by aligning the individual gene sequences of TPI and enolase as a single product. The pTZ57R/T*-enolase* and pGEM-T*-TPI* plasmids encoding the open reading frames of *enolase* (1290 bp) and *TPI* (759 bp), respectively, were constructed. *Enolase* and *TPI* genes were amplified using specific primers from genomic DNA of *L. donovani*. The *TPI* gene was amplified by primers: upstream (5′CATATGATGTCCGCCAAGCCCCAGCCGATC3′) containing a restriction site of NdeI and downstream (5′GGATCCCTTACGAGTGGCATCAATGATGTC3′) containing BamHI site without a stop codon. PCR was performed using the following condition: initial denaturation at 95°C for 5 min, 30 cycles of denaturation at 95°C for 1 min, annealing at 63°C for 30 sec, extension at 72°C for 1 min 30 sec, and a final extension at 72°C for 10 min. After amplification, TPI was inserted into NdeI and BamHI restriction sites of the cloning vector pGEM-T. The enolase forward primer encoding a 5′ BamHI site (5′GGATCCATGCCGATACGAAAGGTTTACGCC3′) and a 3′ EcoRI site (5′GAATTCTTACGCCCAGCCAGGATAGCCGTA3′) was used to amplify *enolase* from the genomic DNA. The PCR amplified *enolase* gene was cloned into BamHI and EcoRI sites of pTZ57R/T vector and subcloned into the bacterial expression vector pET28a at the same restriction sites. Then, the *TPI* gene was digested from the recombinant pGEM-T-*TPI* plasmid by NdeI and BamHI and was fused in-frame to 5′ end of *enolase* in pET28a plasmid to generate pET28a-*TPI-enolase* (pET28a-*T*-*E*). For clone confirmation, recombinant chimeric plasmids of pET28a-*T-E* were digested by NdeI and EcoRI restriction enzymes. The resulting chimeric construct was transformed into the expression host BL21 (DE3) cells. Freshly transformed colonies containing the pET28a-*TPI-enolase* were employed for the recombinant chimeric protein (rLdT-E) purification using LB broth containing kanamycin (50 *μ*g/ml), induced by 0.5 mM IPTG at 37°C for 4 hr. Bacteria were harvested by centrifugation at 8000 × g for 10 min at 4°C, and the protein expression was assessed by 12% SDS-PAGE followed by Coomassie blue staining. Further, the separation of insoluble and soluble protein fractions demonstrated that the majority of rLdT-E chimeric protein was present in the insoluble fraction and the protein was purified from inclusion bodies [25]. Briefly, 500 ml culture was harvested through multiple rounds of centrifugation (8000 × g, 10 min, 4°C), and the supernatant was discarded. The pellet after sonication was washed once with 1% Triton-X-100 followed by sample preparation (1 ml) in 5x Laemmli buffer. Samples were resolved on 12% polyacrylamide gels followed by negative staining with 4 M sodium acetate. The rLdT-E chimeric protein appeared as a transparent band against an opaque background which was excised followed by electroelution in 1 mM EDTA buffer and concentrated using centricons (Millipore USA), dialyzed against PBS. The endotoxin level was measured using a Limulus amoebocyte lysate test (Pierce LAL Chromogenic Endotoxin Quantitation Kit, Thermo Fisher, USA) following the manufacturer's instructions. The purity of the protein was assessed by SDS-PAGE and the concentration quantified through the Bradford assay using bovine serum albumin as a standard.

### 2.3. rLdT-E Expression Validation by Western Immunoblotting

The chimeric protein was separated on a 12% SDS-PAGE gel and transferred onto a PVDF membrane by electrotransfer at 80 mA for 2-3 hours using the tris-glycine buffer. The PVDF membrane containing protein was blocked with 5% skimmed milk for 1 hr at room temperature, followed by incubation in rabbit anti-enolase (1 : 10000) polyclonal antibody for overnight at 4°C. The membrane was then washed with PBS containing 0.05% of Tween-20 (PBST) for 5 times and incubated in anti-rabbit HRP conjugated secondary antibody (1 : 10000) for 1 and 1/2 hr. The membrane was again washed 5 times with PBST followed by the addition of chemiluminescence substrate (Millipore USA), and signals were captured in ChemiDoc (GE Healthcare). Further, MS-MS analysis was carried out to determine the purity of the rLdT-E protein, and the result was interpreted using Mascot database search.

### 2.4. Estimation of Nitric Oxide, Nitric Oxide Synthase (NOS2), and Th1 Cytokines in *Leishmania*-Infected Hamster Macrophages

Intracellular generation of nitric oxide (NO) was estimated in hamsters' peritoneal macrophages using fluorescent probe DAF-2-DA (Sigma-Aldrich, USA) through flow cytometry (Calibur, Becton-Dickinson, USA). For this, 1 × 10^6^ macrophages/ml were seeded in complete RPMI-1640 in 12-well cell culture plates followed by infection with *L. donovani* stationary phase promastigotes at a ratio of 1 : 10 for 12 h at 37°C in a CO_2_ incubator [26]. After the incubation, noninternalised parasites were removed by washing 2-3 times with incomplete RPMI-1640 medium. The infected macrophages were then treated for 48 h with the rLdT-E at variable concentrations (100, 50, 10, and 1 *μ*g/ml), and untreated cells were kept as a negative control. Lipopolysaccharide (LPS; 10 *μ*g/ml, Sigma-Aldrich, USA)-treated cells served as the positive control. After the treatment, the adherent macrophages were detached, washed, and resuspended in chilled PBS and stained with the fluorophore DAF-2DA (2 *μ*M) for 30 min at 37°C, and fluorescence was acquired in Calibur using CellQuest software. The data were expressed in the form of median fluorescence intensity (MFI). The mRNA expression of NOS2 and Th1 cytokines, interferon-*γ* (IFN-*γ*) and Tumor Necrosis Factor-*α* (TNF-*α*), was measured through quantitative RT-PCR. In brief, RNA from uninfected and infected peritoneal macrophages (1 × 10^6^ cells/well/ml in a 12-well plate) treated with or without chimeric protein (at 48 h poststimulation) was extracted using a RNeasy mini isolation kit (Qiagen, Germany). RNA samples were quantified in NanoDrop (Thermo Fischer Scientific, USA), and cDNA were synthesized using a High-Capacity cDNA Reverse Transcription Kit (Thermo Scientific, USA). RT-PCR of these cDNA samples was performed in an iQ5 multicolor real-time PCR detection system (Bio-Rad) using the following reaction conditions: initial denaturation at 95°C for 2 min followed by 40 cycles, each consisting of denaturation at 95°C for 20 s, annealing at 60°C for 20 s, and extension at 72°C for 16 s per cycle employing various sets of primers designed with the help of beacon designer software as listed in Table 1. The relative fold changes in the mRNA expressions of different cytokines were estimated by the 2^−*ΔΔ*Ct^ method by comparing them to the RPL18 gene [27].

### 2.5. *In Vitro* Evaluation of Proliferative Effect of rLdT-E Stimulation on Lymph Node Cells

To assess the cellular response generated by chimeric protein, a lymphocyte proliferation assay was performed according to the Garg et al. protocol [23]. Lymphocytes (1 × 10^6^ cells/ml) from naive/infected/treated hamsters were incubated in complete RPMI-1640 (Sigma-Aldrich, USA). 0.1 ml of cell suspension was seeded in 96-well flat-bottom tissue culture plates (Nunc, Denmark) and stimulated with the recombinant chimeric protein (rLdT-E) and SLD as the control at a concentration of 10 ng/ml in triplicate. Concanavalin A (ConA, 10 ng/ml, Sigma-Aldrich, USA) was used as a standard mitogen. Wells without stimulants served as blank controls. Cells were maintained in a CO_2_ incubator (37°C with 5% CO_2_) for 72 h. After incubation, 100 *μ*l of culture suspension was aspirated from all wells, and subsequently, 50 *μ*l of XTT (Biological Industries, Israel) was added to the remaining 100 *μ*l of culture supernatant around 4 h prior to termination of the experiment. The absorbance of samples was measured at 480 nm with 650 nm as a reference wavelength in a SpectraMax Plus 384 microplate reader (Molecular Devices, USA).

### 2.6. Evaluation of Therapeutic Efficacy of rLdT-E Adjuvanted with BCG in *L. donovani*-Infected Hamsters

#### 2.6.1. Immunotherapy Schedule

In the first set of experiments, hamsters (*n* = 7 per group) were infected with 5 × 10^5^ amastigotes *via* the i.c. route (needle length 13 mm) except group I (naïve control), and fifteen days p.i., group III, IV, V, and VI animals were vaccinated with 10 *μ*g, 25 *μ*g, 50 *μ*g, and 100 *μ*g of rLdT-E protein, respectively, *via* i.d. route (needle length 8 mm) thrice at fifteen days' intervals at the similar respective concentrations. Group II animals were considered as an infected control. On day 60 p.i., all experimental animals were sacrificed for the assessment of parasitic burden.

In a separate set of experiments, a total of 48 hamsters divided into 4 groups (*n* = 12) were used wherein groups I and II were considered naive and infected controls, respectively. Animals of all groups except group I were infected with 5 × 10^5^ amastigotes, and fifteen days p.i., group III animals were injected i.d. with the adjuvant BCG, i.e., Bacillus Calmette-Guerin Moscow strain 361-I (Tubervac) (a live freeze-dried vaccine derived from an attenuated strain of *Mycobacterium bovis*) at a dose of 2 × 10^5^ C.F.U./100 *μ*l/animal. For immunotherapy, group IV animals were vaccinated with 100 *μ*g of rLdT-E protein in combination with BCG. A similar dosing schedule was followed twice after the first prime dosing at two weeks' intervals in groups III and IV. The immunotherapeutic efficacy of the rLdT-E along with BCG was determined through parasite burden and immunological responses by sacrificing six hamsters from each group on days 60 and 90 p.i.

#### 2.6.2. Assessment of Parasite Burden, Delayed-Type Hypersensitivity, and Lymphoproliferative Response

Parasite load was represented as the number of amastigotes per 100 macrophage cell nuclei in Giemsa-stained splenic impression smears. Delayed-type hypersensitivity (DTH) response was estimated in terms of percentage increase in footpad thickness in response to SLD antigen on the contralateral footpad measured after 48 h as per the protocol by Kumari et al. [17]. The lymphoproliferative response was evaluated in mononuclear lymph node cells of all animals in experimental groups at both days 60 and 90 p.i. as per the method described previously [23].

#### 2.6.3. Estimation of Th1 and Th2 Cytokine Response

Relative fold change in the expression of cytokine mRNA transcripts was estimated in their splenic tissues through quantitative real-time PCR (iQ5 LightCycler, Bio-Rad) using similar reaction conditions as mentioned in Section 2.4, employing various sets of Th1 (IFN-*γ*, IL-12, and TNF-*α*) and Th2 (IL-10, IL-4, and TGF-*β*) cytokine primers designed with the help of beacon designer software as listed in Table 1. In addition, the levels of Th1 and Th2 cytokines were estimated in animals' sera by ELISA following kit specifications (Krishgen ELISA kit, India).

#### 2.6.4. Analysis of Antibody Response

Sera were collected on days 60 and 90 p.i., and the specific serum IgG isotype antibody response was measured by ELISA using a reagent set from BD, San Diego, following the protocol of Garg et al. [23]. The concentration of antigen, titer of test, and normal sera samples (primary antibody) as well as each lot of the conjugates (secondary antibodies) were subjected to checkerboard titration to define optimal dilution [28]. Briefly, 96-well microtiter plates (Nunc MaxiSorp; eBioscience) were coated with SLD antigen (10 *μ*g/ml) and rLdT-E protein (5 *μ*g/ml) in coating buffer separately and incubated overnight at 4°C. After 3 washes with washing buffer, wells were blocked with 200 *μ*l/well assay diluent (BD, San Diego) for 2 h at 37°C. Further, 100 *μ*l/well of diluted serum samples (1 : 200) in duplicate was added followed by incubation at 37°C for 2 h and washed again 5 times. Wells were incubated with 100 *μ*l/well biotin-conjugated mouse anti-hamster IgG1 and IgG2 (1 : 1000) for 2 h at 37°C. Subsequently, after washing, 100 *μ*l of horseradish peroxidase- (HRP-) conjugated streptavidin (1 : 1000) was added and incubated for another 1 h at 37°C. The enzyme-labelled complexes were detected by incubation with substrates A and B mixed in a 1 : 1 ratio (100 *μ*l/well) for 15-20 min. The reaction was stopped using stop solution, and optical density at 492 nm was determined in a SpectraMax Plus 384 microplate reader (Molecular Devices, USA).

### 2.7. Statistical Analysis

Data analysis was performed with GraphPad Prism 8 (GraphPad Software, San Diego, CA, USA). Statistical significance between different independent groups was determined by Student's *t*-test and ordinary two-way analysis of variance tests. Two sets of experiments were performed for the vaccination studies, and the results are expressed as the mean ± SEM with a *p* value of ≤0.05 considered significant.

## 3. Results

### 3.1. Expression and Purification of rLdT-E (TPI-Enolase) Chimeric Protein

**Figure 1:**
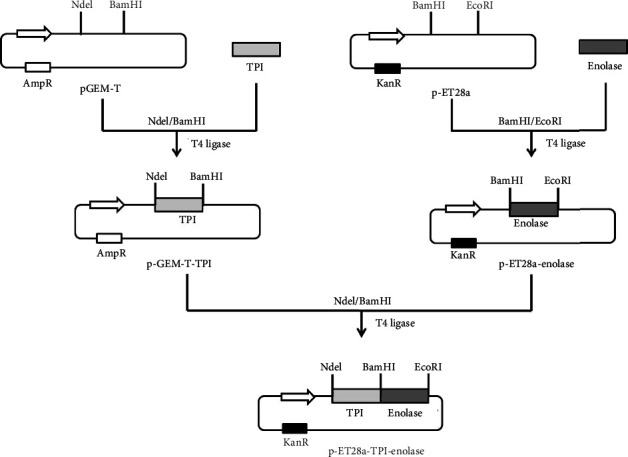
Generation of chimera of *TPI* and *enolase*. Schematic representation of cloning of chimeric construct in pET28a vector.

**Figure 2:**
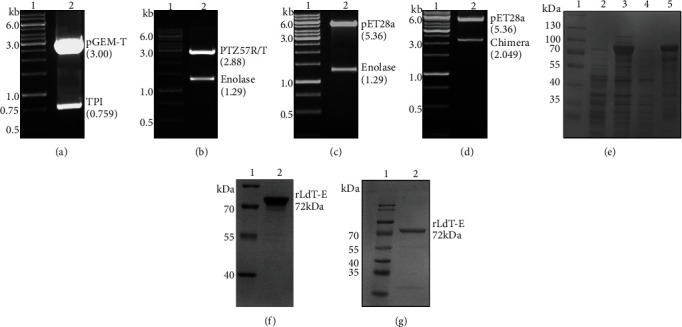
Cloning and expression of rLdT-E chimeric protein. In (a–d), lane 1 is the DNA ladder. (a) Restriction digestion of *TPI* insert (0.759 kb) cloned in pGEM-T cloning vector (3 kb) with NdeI and BamHI (lane 2). (b) Restriction digestion of *enolase* insert (1.29 kb) cloned in pTZ57R/T cloning vector (2.88 kb) with BamHI and EcoRI (lane 2). (c) Restriction digestion of *enolase* insert (1.29 kb) cloned in pET28a(+) expression vector (5.36 kb) with BamHI and EcoRI (lane 2). (d) Restriction digestion of chimera insert (2.049 kb) cloned in pET28a(+) expression vector with NdeI and EcoRI (lane 2). Purification of rLdT-E chimeric protein. In (e–g), lane 1 is the protein molecular weight marker. Expression of rLdT-E was induced by 0.5 mM IPTG in transformed BL21 (DE3) cells harboring pET28a-T-E. (e) Total bacterial lysate was analyzed on SDS-PAGE stained with Coomassie blue. Uninduced and induced samples in lane 2 and lane 3, respectively; lane 4 is soluble fraction, and lane 5 denotes insoluble fraction. (f) Purified rLdT-E chimeric on SDS-PAGE gel (lane 2). (g) Immunoblot analysis of purified rLdT-E chimeric protein probed by the anti-enolase antibody (lane 2).

Two *L. donovani* antigens, TPI and enolase, were genetically linked in tandem to generate a single recombinant chimeric protein, and its cloning strategy is described well in Figure 1. The veracity of all resultant plasmids was confirmed by restriction digestion (Figures 2(a)–2(d)). The length of the predicted ORF is 2049 nucleotides coding for a polypeptide of molecular weight ~72 kDa. Expression of rLdT-E chimeric protein was induced by using 0.5 mM IPTG at 37°C (Figure 2(e)) and purified from inclusion bodies (Figure 2(f)). Further, the purified chimeric protein was validated by a western blot assay using previously raised rabbit anti-enolase antibody on the immunoblot as demonstrated in Figure 2(g). The rLdT-E protein was also validated by MALDI-TOF MS/MS. The LPS content of the rLdT-E was measured by a Limulus amoebocyte lysate test (Pierce LAL Chromogenic Endotoxin Quantitation Kit, Thermo Scientific) and found to be below 10 endotoxin units (EU)/mg.

### 3.2. rLdT-E Induced NOS2 and Th1 Cytokine Expression in Infected Macrophages and Triggers Lymphocyte Proliferation in response to Antigenic Stimuli

**Figure 3:**
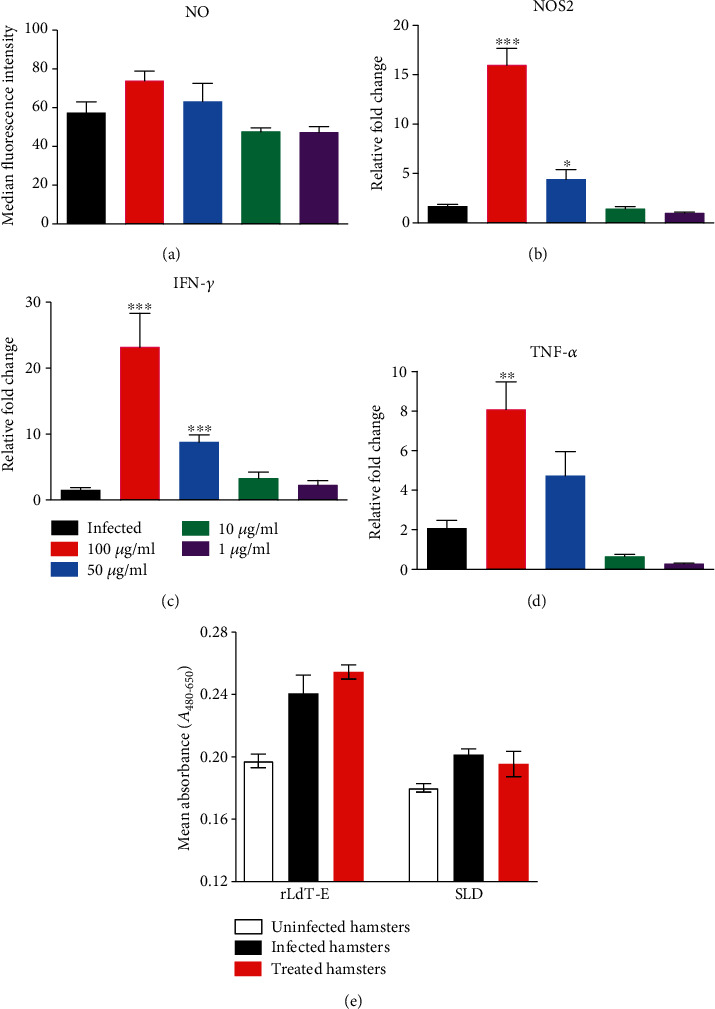
(a) Generation of NO in *Leishmania*-infected macrophages stimulated with rLdT-E chimeric protein (1, 10, 50, and 100 *μ*g/ml) as compared to the unstimulated infected cells. (b–d) Relative fold change in the mRNA expression of NOS2 and Th1 cytokines (IFN-*γ* and TNF-*α*) in infected peritoneal macrophages stimulated with rLdT-E in variable doses (1, 10, 50, and 100 *μ*g/ml) as compared to unstimulated cells. (e) Lymphoproliferative response in lymph node cells of *Leishmania*-infected and treated hamsters upon stimulation with rLdT-E protein and SLD antigen was used as control (10 ng/ml). Data is represented as the mean ± SEM. Significance values (^∗^*p* ≤ 0.05, ^∗∗^*p* ≤ 0.01, and ^∗∗∗^*p* ≤ 0.001) of stimulated groups were calculated with respect to controls using Student's unpaired *t*-test.

Nitric oxide, a key mediator of nonspecific immunity, generated in host macrophages has been demonstrated to be responsible for the intracellular killing of *Leishmania* parasites [29]. *L. donovani* promastigote-infected macrophages upon stimulation with rLdT-E protein (100 *μ*g/ml) had a marginal increase in NO production (Figure 3(a)) after 48 h of stimulation as compared to the infected unstimulated cells, indicating towards its leishmanicidal activity. This observation was further supported by significantly enhanced mRNA expression of NOS2, IFN-*γ*, and TNF-*α* in rLdT-E-stimulated cells (Figures 3(b)–3(d)). LPS-treated and unstimulated cells served as positive and negative controls, respectively. The cellular response elicited by lymphocytes isolated from lymph nodes of naive, infected, and treated hamsters was assessed through XTT after stimulation with proteins at a dose of 10 ng/ml (Figure 3(e)). ConA was used to assess the procedural sensitivity. A higher level of proliferative responses in infected and treated hamsters' lymphocytes stimulated with rLdT-E chimeric protein (0.2408 ± 0.01169, 0.2545 ± 0.0046) than the control SLD-stimulated lymphocytes indicates the chimera's immunogenicity against *Leishmania* infection.

### 3.3. rLdT-E/BCG Immunotherapy Leads to a Marked Reduction in Splenic Parasite Load

**Figure 4:**
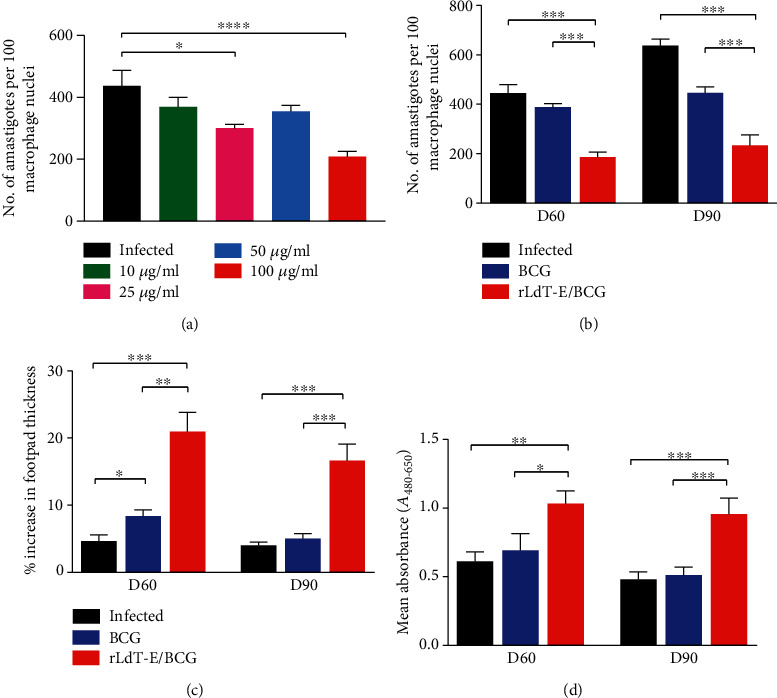
(a) Parasite load expressed as the number of amastigotes per 100 macrophage nuclei in splenic dab smears of rLdT-E protein (10, 25, 50, and 100 *μ*g/hamster) treated and infected hamsters. Significance values indicate the difference between the treated (*n* = 7/group) and infected (*n* = 7) groups. (b) Parasite load in splenic dab smear of rLdT-E/BCG-treated animals, (c) DTH response to SLD as % increase in footpad thickness, and (d) lymphoproliferative response in treated animals against SLD in comparison to the untreated infected animals on days 60 and 90 p.i. Student's unpaired *t*-test and ordinary one-way ANOVA (Dunnett test) were used to calculate the statistical significance between the treated and untreated groups (^∗^*p* ≤ 0.05, ^∗∗^*p* ≤ 0.01, and ^∗∗∗^*p* ≤ 0.001). Data represents the mean ± SEM of two independent experiments with similar results.

The rLdT-E protein's efficacy was evaluated at different concentrations which revealed that 100 *μ*g was the most efficacious dose among all tested doses as characterized by moderate but significant ~52% reduction in the splenic parasite load (Figure 4(a)). Further, to assess the immunotherapeutic potential of rLdT-E protein with an immunomodulator, amastigote-infected animals were treated with a 100 *μ*g dose of rLdT-E in combination with BCG. A significant reduction in parasite load was observed in the rLdT-E/BCG-treated group (70% inhibition) over infected and BCG alone-treated groups on day 60 p.i. which reduces to 65% by day 90 p.i. Animals treated with BCG alone did not confer any noticeable reduction in burden over untreated controls and showed an increased number of parasites at the later time point as demonstrated in Figure 4(b).

### 3.4. Treatment with rLdT-E/BCG Elevated Intradermal Response and Lymphocyte Proliferation after Stimulation with *L. donovani* Antigen

Though significantly (*p* ≤ 0.001) enhanced DTH response was noticed in animals vaccinated with rLdT-E/BCG at both time points, the response was more pronounced on day 60 p.i. indicating a slight waning of the response over time. The BCG-treated group also showed a partial enhancement (*p* ≤ 0.05) in footpad swelling in comparison to the untreated infected group earlier, but no such response was observed later on (Figure 4(c)). Nonetheless, a remarkable increase in lymphocyte proliferation was observed in the rLdT-E/BCG-treated group on days 60 (1.033 ± 0.09275) and 90 (0.9547 ± 0.1169) p.i. in comparison to both controls, which was consistent with the DTH response. However, BCG control animals showed only a marginally enhanced lymphoproliferative response on both time points (0.6936 ± 0.1204 on day 60 p.i. and 0.5124 ± 0.05851 on day 90 p.i.) which might be the underlying cause of a diminished reduction in parasite burden in this particular group (Figure 4(d)).

### 3.5. rLdT-E/BCG Vaccination Favored the Dominance of Th1 Phenotype as Determined through Cytokine mRNA Expression Analysis

**Figure 5:**
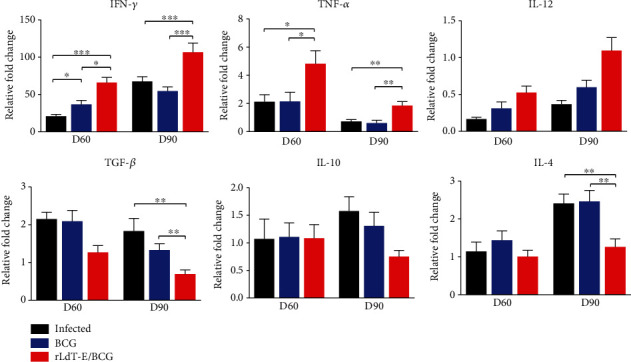
Splenic cytokine mRNA expression profile of infected and vaccinated animals on days 60 and 90 p.i. by quantitative real-time PCR. Student's unpaired *t*-test was used to calculate the statistical significance between the treated and untreated groups (^∗^*p* ≤ 0.05, ^∗∗^*p* ≤ 0.01, and ^∗∗∗^*p* ≤ 0.001). Data represents the mean ± SEM of two independent experiments with similar results.

Among Th1 cytokines, IFN-*γ* was significantly (*p* ≤ 0.05) increased in the rLdT-E/BCG-treated group on day 60 p.i. over untreated infected control which increased progressively by day 90 p.i. (*p* ≤ 0.001) in comparison to both controls. Similarly, TNF-*α* expression was also increased significantly (*p* ≤ 0.05) though the expression was lowered at a later time point in all groups. Moreover, marginally increased IL-12 was also noticed in this group on day 60 p.i., which gradually increases by day 90 p.i.; however, the change was insignificant. No noteworthy change in Th1 cytokine expression was observed in animals treated with BCG alone, except a partial (*p* ≤ 0.05) increase in IFN-*γ* expression. On the contrary, an apparent decrease in TGF-*β* and IL-4 was evident in the rLdT-E/BCG-treated group on day 60 p.i. which became significant (*p* ≤ 0.01) by day 90 p.i. in relation to both controls. The mRNA expression of IL-10 which was observed to be similar in all experimental groups on day 60 p.i. partially decreased at the later time point in the rLdT-E/BCG-treated group as compared to the infected control. Meanwhile, the BCG alone-treated group was not showing any remarkable change in the expression of Th2 cytokines except a marginal downregulation in TGF-*β* on day 90 p.i. (Figure 5).

### 3.6. rLdT-E/BCG Vaccination Induced the Production of Th1-Associated Cytokines in Sera

**Figure 6:**
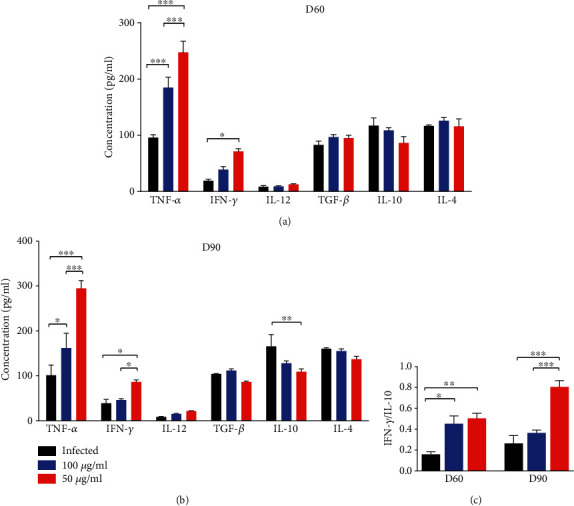
Cytokine production in rLdT-E-treated hamsters in comparison to BCG-treated and infected hamsters on days (a) 60 and (b) 90 p.i. and (c) ratio of IFN-*γ*/IL-10. Significance values (^∗^*p* ≤ 0.05, ^∗∗^*p* ≤ 0.01, and ^∗∗∗^*p* ≤ 0.001) indicate the difference between treated and control groups and were calculated using two-way ANOVA and Student's unpaired *t*-test. Data represents the mean ± SEM of two independent experiments with similar results.

The skewness in the cytokine milieu in response to rLdT-E/BCG vaccination was also estimated in serum samples of treated animals. There was significant (*p* ≤ 0.05) production of IFN-*γ* in the rLdT-E/BCG-treated group on day 60 p.i. (62.30 ± 6.934 pg/ml), and the level gradually increased by day 90 p.i. (86.27 ± 4.425 pg/ml) in comparison to untreated infected and BCG controls. However, the level of IFN-*γ* which was found to be slightly higher (48.64 ± 7.713 pg/ml) in the BCG control at an early time point became almost equivalent to that of the infected control by day 90 p.i. Although a significant (*p* ≤ 0.001) increase in TNF-*α* was observed in the rLdT-E/BCG-treated as well as BCG alone-treated groups at both time points with respect to infected control animals, the response was more prominent in the rLdT-E/BCG group (294.6 ± 17.48 pg/ml). There was a partial production of IL-12 cytokine in sera of the rLdT-E/BCG group (21.43 ± 0.5551 pg/ml) as compared to both controls on day 90 p.i. Conversely, levels of TGF-*β* and IL-4 have decreased apparently in the rLdT-E/BCG-treated animals at the later time point, and IL-10 (109.2 ± 6.634 pg/ml) decreased significantly (*p* ≤ 0.01) on day 90 p.i. in this group in comparison to untreated infected animals (140.3 ± 2.566). Moreover, the rLdT-E/BCG also induced significantly higher IFN-*γ*/IL-10 ratios at the early observation point which increases by day 90 p.i. indicating the polarized Th1 response generated by the chimeric protein (Figure 6).

### 3.7. rLdT-E/BCG Stimulated Parasite and Antigen-Specific Humoral Response

**Figure 7:**
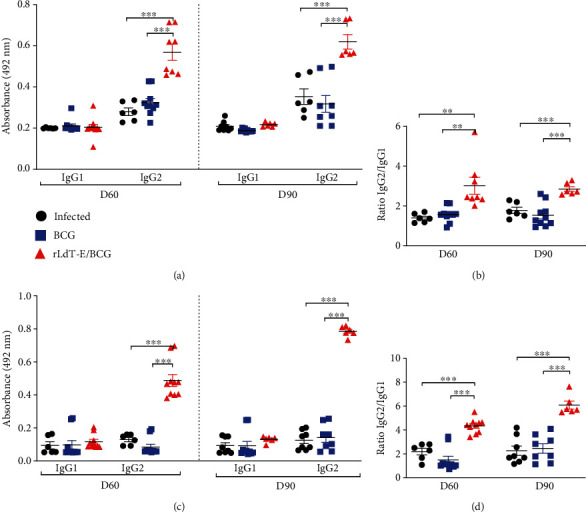
Humoral reactivity against SLD antigen and rLdT-E on days 60 and 90 p.i. Comparison between IgG1 and IgG2 antibodies against SLD antigen (a) and rLdT-E. (b) Ratio of IgG2/IgG1 against SLD antigen (c) against rLdT-E (d). Significance values (^∗^*p* ≤ 0.05, ^∗∗^*p* ≤ 0.01, and ^∗∗∗^*p* ≤ 0.001) indicate the difference between treated and control groups and calculated using Student's unpaired *t*-test. Data represents the mean ± SEM of two independent experiments with similar results.

Unlike mice, in hamsters, there are no distinct classifications of Ig, and it is believed that hamster IgG1 corresponds to mouse IgG1 and hamster IgG2 corresponds to mouse IgG2a/IgG2b. It is well known that a high level of IgG2 antibodies depicts a Th1-biased response while IgG1 represents the Th2 phenotype [30]. In VL, antibody levels correlate with intensity of infection harbored by the host. We characterized the anti-Leishmania and anti-rLdT-E humoral responses in the animals' serum samples by analyzing IgG1 and IgG2 antibodies in the current study to determine the inclination of T-cell response either towards protection or disease progression. The humoral reactivity against parasite antigen showed that animals treated with rLdT-E/BCG exhibited a highly significant (*p* ≤ 0.001) increase in *Leishmania*-specific IgG2 antibodies in comparison to controls at both the observation points, more prominent on day 90 p.i. Moreover, the ratio of IgG2/IgG1 was also shown to be significant (~2-fold) in the rLdT-E/BCG-treated group on both time points. On the other hand, the IgG1 level was almost equivalent in all experimental groups, showing no significant difference. A marked increase (*p* ≤ 0.001) was observed in the anti-protein IgG2 level along with significantly (*p* ≤ 0.001) higher IgG2/IgG1 ratios in the rLdT-E/BCG-treated animals which strongly supported the chimeric protein's immunogenicity as well as the inclination of immune responses towards Th1 phenotype (Figure 7). Both control groups, however, had lower levels of antiparasite and anti-rLdT-E IgG2 responses, corroborating with the Th2 response in these animals. Collectively, it can be inferred that rLdT-E in association with BCG stimulates both parasite and antigen-specific antibody responses, indicating the generation of protective immunity upon rLdT-E/BCG treatment.

## 4. Discussion

The development of a highly effective and durable vaccine has become a key priority for controlling VL. Recent innovations and decades of endeavor resulted in a diverse pipeline of novel vaccine candidates against VL ranging from traditional live vaccines to advanced recombinant polyprotein and multiantigenic T-cell epitope-based vaccines, in preclinical and clinical trials; however, translation of these to develop a human administrable vaccine for VL is still arduous. Therapeutic vaccines alone or in combination with potential immunomodulator cause reestablishment of the natural immunity in the infected host, and with this understanding, in this study, two potential prophylactic vaccine candidates, enolase and TPI [18, 19], were fused as a single chimeric construct to evaluate its immunogenicity and immunotherapeutic efficacy against *L. donovani* infection. Inclusion of multiple antigens into a vaccine for prophylactic/therapeutic application presents a way to circumvent differing immune recognition within a heterogeneous population besides broad coverage of antigenic diversity of parasite and augmented immune response in the host polymorphic system [8]. The rLdT-E chimeric protein thus generated was assessed for its potentiality as a vaccine antigen, and increased *in vitro* lymphocyte proliferative responses in *Leishmania*-infected as well as treated hamsters were observed upon stimulation with the chimeric protein as compared to the control SLD antigen, thus substantiating its immunogenicity against *Leishmania* infection [31, 32]. Although a lymphoproliferative assay indicates T-cell function, the generation of immunostimulatory response in macrophages could be an additional criterion to judge the ability of any vaccine candidate in evoking immune response against the parasite. Subsequently, experiments performed herein demonstrated that the rLdT-E chimeric protein leads to a significantly enhanced mRNA expression of NOS2 with a partially increased intracellular NO level and an anti-Leishmanial effector molecule post *L. donovani* infection. This finding was further supported by enhanced mRNA expression of Th1 cytokines, *viz.*, IFN-*γ* and TNF-*α* [33], indicating the ability of rLdT-E to induce innate immune responses against *Leishmania* in an NO-dependent manner and exert host-protective immunoregulatory effects [34–39]. Therefore, these outcomes pave the way for the evaluation of the immunotherapeutic potential of rLdT-E chimeric protein in *Leishmania*-infected hamsters. Since the dose of an antigen is one of the important factors in determining the outcome of the immune responses, infected hamsters were treated with variable doses of rLdT-E protein [40]. The maximum reduction in the parasite burden observed in hamsters treated with the highest dose of 100 *μ*g (~52%, ^∗∗∗^*p* ≤ 0.001) was in agreement with Santos and Aguiar [41], who also used a high dose of 150 *μ*g of protein FML to treat *L. donovani*-infected BALB/c mice and observed a remarkable reduction in parasite load. As reported, due to lower immunogenicity, recombinant protein-based vaccines require an appropriate immunostimulatory molecule for the requisite induced immune response [11]. Hence, efforts were made to augment the efficacy of the chimera by combining rLdT-E with a potent adjuvant, BCG, which has already been employed as an immunostimulatory agent for therapy purposes against cutaneous leishmaniasis, and effectively revealed similar cure rates with reduced treatment duration and side effects than standard anti-Leishmanial treatments [42]. Previously, in some *Leishmania* vaccine trial studies, few unpleasant reactions of BCG adjuvant have been reported in the murine model, Indian langur, and rhesus monkeys [43–45], despite BCG being extensively employed in clinical trials of vaccines against leishmaniasis due to its potential immunomodulatory properties [46]. In accordance, rLdT-E/BCG combination exhibited a considerable immunotherapeutic efficacy with a significant reduction in splenic parasite load on days 60 and 90 p.i., ~70% and 65%, respectively, which was more prominent than the only BCG-treated group. As we observed a considerable efficacy in response to rLdT-E/BCG in animals infected intracardially with a high dose of virulent amastigotes even on the late observation time point, there is a strong possibility that the therapeutic efficacy of the chimeric protein will enhance significantly during field trials where the intradermal route of administration is followed. Since chronic infections are usually characterized by T-cell exhaustion and many vaccination studies emphasize the significance of circulating T-lymphocyte restoration for clinical improvement and control of parasitism, thus, the activation and augmentation of an effective cellular response through immunotherapy could contribute to the treatment of the disease [47, 48]. Consistent with these reports, vaccination with rLdT-E/BCG yielded a positive lymphoproliferative response along with a prominent intradermal DTH response, which is a characteristic of cell-mediated immunity induced by Th1 cells and a desired effect for a possible vaccine candidate [49].

A strong Th1 response offers a rational basis for effective therapeutic intervention, and to prove that the rLdT-E antigen has immunomodulatory effect, we analyzed the alterations in Th1 and Th2 cytokine milieu in splenic tissues and serum from hamsters [50]. Predominantly, Th1 cytokines IFN-*γ*, TNF-*α*, and IL-12 are correlated with protection or natural resistance against VL, wherein IFN-*γ* induces NO production which causes parasite destruction, thereby controlling parasitism through microbicidal activity [51]. Similarly, TNF-*α* synergize with IFN-*γ* in parasite killing [52], and IL-12 plays a pivotal role in the development of Th1-associated immunity as it induces IFN-*γ* production and allows differentiation and proliferation of naive T-cells into Th1 cells [53]. Confirming that, in the present investigation, a significantly elevated level of IFN-*γ* and TNF-*α* in splenic tissues as well as in sera of rLdT-E/BCG-treated animals provided correlates of disease resistance offering a good indication of the achieved vaccine's efficacy [53]. In addition, an elevated IFN-*γ*/IL-10 ratio in response to the chimeric protein treatment reflects the Th1-driven immune polarization in corroboration with the above findings [54]. On the contrary, Th2 cytokines, TGF-*β* and IL-4, which play a critical role in parasite survival, disease progression, and suppression of Th1-associated cure of murine VL [52, 55] were decreased in rLdT-E/BCG-treated animals' splenic tissues, and a noticeable decrease in the immunosuppressive cytokine, IL-10, was also observed in sera of treated animals. Hence, the ability of rLdT-E/BCG to elicit such Th1-inclined responses even after the establishment of infection endorses its potential immunotherapeutic effect to generate curative responses which was consistent with the development of protective immunity against the fatal VL infection [56]. Besides, the immunotherapy treatment with the rLdT-E/BCG vaccine candidate also conferred considerable enhancement in anti-*Leishmania* and anti-rLdT-E-specific IgG2 further supporting the remarkably enhanced cellular response and reduced parasitic burden [54, 56, 57]. The magnitude of the antibody response in rLdT-E/BCG vaccinated animals signifies the induction of protective/curative Th1 response also observed in the case of commercialized canine VL vaccines [57, 58].

Since the majority of inhabitants in disease-endemic regions are asymptomatic serving as a potential reservoir which may develop into full-blown clinical VL [2] and current drugs are too toxic to justify their use in these otherwise healthy individuals, a therapeutic vaccine is in dire need to tackle this scenario. The current study is a way forward to meet this goal. rLdT-E as a potential therapeutic chimeric vaccine candidate could target the *Leishmania*-infected population by boosting their immunity and lowering the gross parasitic load in the endemic population.

In a nutshell, our findings highlight the apparent immunostimulatory property of the rLdT-E chimera in infected macrophages along with its therapeutic efficacy when adjuvanted with BCG in reducing the severity of infection, well-substantiated through robust Th1-inclined cellular and humoral immune responses against *L. donovani*. To our knowledge, this is the first report showing the potentiality of the therapeutic chimeric vaccine candidate comprising two glycolytic proteins against human VL. Efforts are currently underway to enhance the therapeutic potential of this novel chimera by employing other potent immunomodulators like GLA-SE, saponin which was allied with stimulating Th1-biased immune response and optimizing the treatment regimen [9, 59], thereby fortifying the host immunity proficiently enough to resist the dreaded Kala-azar disease. Future studies will explore the ability of the recombinant chimeric protein for its transition from preclinical testing to further trials.

## Figures and Tables

**Table 1:** Sequences of forward and reverse primers of hamster cytokines used for real-time quantitative PCR (RT-qPCR).

Genes	Direction	Primer sequence	Product length
RPL 18	Forward	5′ AACTCCACCTTCAATCAG 3′	96
	Reverse	5′ GATGATCCGAAAGATGAAG 3′	
NOS2	Forward	5′ TGCCTTGCATCCTCATTGG 3′	77
	Reverse	5′ GTCGCTGTTGCCAGAAACTG 3′	
TNF-*α*	Forward	5′ GGAGTGGCTGAGCCATCGT 3′	131
	Reverse	5′ AGCTGGTTGTCTTTGAGAGACATG 3′	
IFN-*γ*	Forward	5′ GCTTAGATGTCGTGAATGG 3′	200
	Reverse	5′ GCTGCTGTTGAAGAAGTTAG 3′	
IL-12	Forward	5′ AATTACTCTGGACGGTTCAC 3′	81
	Reverse	5′ GCTACTGCTGCTCTTGAC 3′	
TGF-*β*	Forward	5′ ACGGAGAAGAACTGCTGTG 3′	178
	Reverse	5′ GGTTGTGTTGGTTGTAGAGG 3′	
IL-4	Forward	5′ CCACGGAGAAAGACCTCATCTG 3′	75
	Reverse	5′ GGGTCACCTCATGTTGGAAATAAA 3′	
IL-10	Forward	5′ GTTGCCAAACCTTATCAGAAATGA 3′	102
	Reverse	5′ TTCTGGCCCGTGGTTCTCT 3′	

## Data Availability

All the data supporting the results of the current study is available with the authors.
